# The neighborhood context and all-cause mortality among older adults in Puerto Rico

**DOI:** 10.3389/fpubh.2023.995529

**Published:** 2023-03-09

**Authors:** Catherine García, Marc A. Garcia, Mary McEniry, Michael Crowe

**Affiliations:** ^1^Department of Human Development and Family Science, Aging Studies Institute, Center for Aging and Policy Studies, and the Lerner Center for Public Health Promotion and Population Health, Syracuse University, Syracuse, NY, United States; ^2^Department of Sociology, Aging Studies Institute, Center for Aging and Policy Studies, and the Lerner Center for Public Health Promotion and Population Health, Syracuse University, Syracuse, NY, United States; ^3^Center for Demography and Ecology, and Center for Demography of Health and Aging, University of Wisconsin-Madison, Madison, WI, United States; ^4^University of Alabama at Birmingham, Birmingham, AL, United States

**Keywords:** neighborhood characteristics, mortality, Puerto Rican adults, latent variable analysis, multilevel survival analysis, PREHCO, older adults, social determinants of health (SDOH)

## Abstract

**Background:**

Recent efforts have been made to collect data on neighborhood-level attributes and link them to longitudinal population-based surveys. These linked data have allowed researchers to assess the influence of neighborhood characteristics on the health of older adults in the US. However, these data exclude Puerto Rico. Because of significantly differing historical and political contexts, and widely ranging structural factors between the island and the mainland, it may not be appropriate to apply current knowledge on neighborhood health effects based on studies conducted in the US to Puerto Rico. Thus, we aim to (1) examine the types of neighborhood environments older Puerto Rican adults reside in and (2) explore the association between neighborhood environments and all-cause mortality.

**Methods:**

We linked data from the 2000 US Census to the longitudinal Puerto Rican Elderly Health Conditions Project (PREHCO) with mortality follow-up through 2021 to examine the effects of the baseline neighborhood environment on all-cause mortality among 3,469 participants. Latent profile analysis, a model-based clustering technique, classified Puerto Rican neighborhoods based on 19 census block group indicators related to the neighborhood constructs of socioeconomic status, household composition, minority status, and housing and transportation. The associations between the latent classes and all-cause mortality were assessed using multilevel mixed-effects parametric survival models with a Weibull distribution.

**Results:**

A five-class model was fit on 2,477 census block groups in Puerto Rico with varying patterns of social (dis)advantage. Our results show that older adults residing in neighborhoods classified as *Urban High Deprivation* and *Urban High-Moderate Deprivation* in Puerto Rico were at higher risk of death over the 19-year study period relative to the *Urban Low Deprivation* cluster, controlling for individual-level covariates.

**Conclusions:**

Considering Puerto Rico's socio-structural reality, we recommend that policymakers, healthcare providers, and leaders across industries to (1) understand how individual health and mortality is embedded within larger social, cultural, structural, and historical contexts, and (2) make concerted efforts to reach out to residents living in disadvantaged community contexts to understand better what they need to successfully age in place in Puerto Rico.

## 1. Introduction

The twenty-first century in the Commonwealth of Puerto Rico (hereafter, Puerto Rico)—an unincorporated United States (US) territory—is an era characterized by rapid population aging, reductions in social and economic resources, rampant disparities in access to adequate healthcare, and the ongoing reconstruction of the built environment post-Hurricanes Irma and María (1–6). The constellation of these factors infers that many older Puerto Rican adults may lack access to resources, services, and contexts considered necessary for promoting healthy aging.^1^ In order to understand contemporary conditions in Puerto Rico, it is important to consider how historical contexts contribute to health inequities over time, particularly for older adults at increased risk for poor health and mortality.

Researchers have argued that social, political, and economic inequalities in Puerto Rico derive from the impacts of US colonialism—a structural and social determinant of health (7, 8). One significant impact of US colonialism was the transition of Puerto Rico from a rural agricultural society to an urban industrial society in the early twentieth century (9). This transition brought public health benefits, including improved sanitation practices and housing conditions, the creation of local health boards and hospitals, and increased access to primary education. However, urbanization in Puerto Rico also led to widening economic and racial disparities that resulted in unfavorable neighborhood and living conditions among socially marginalized individuals (e.g., poor and Black Puerto Ricans) (10).

For example, San Juan, the capital of Puerto Rico, has a long history of continuous urban growth and economic development. Under US control, San Juan experienced substantial modernization, including changes in land use efficiency and aggregation of local areas that connected land use with global-scale factors. Notably, new and growing opportunities in the San Juan wage labor market were a major driver for rural-dwelling Puerto Ricans to relocate in the early twentieth century; this rural-to-urban migration affected the subsequent development and preservation of several neighborhoods in the metropolitan area (11). Due to their lower socioeconomic position, Puerto Ricans from rural areas were forced to reside in poor and disadvantaged, communities in San Juan, such as La Perla (12). In addition to rural-urban migration patterns, rapid population growth and efforts to mirror the US model of suburbanization were additional factors that influenced variations in the investment of resources across neighborhoods in San Juan throughout the twentieth century that contributed to contemporary residential segregation patterns (13). For example, a study examining residential segregation in the San Juan-Bayamón metropolitan area, the most racially diverse metropolitan area in Puerto Rico, found that neighborhoods with a higher percentage of Black residents were associated with lower socioeconomic status (14).

In addition, a study focusing on the socioeconomic features of neighborhoods to assess health disparities in Puerto Rico found that municipalities (considered county-equivalents by the US Census) with a low socioeconomic position (SEP) were linked to higher cancer-related mortality rates (15). Importantly, the study showed that more deprived municipalities of Puerto Rico were in the island's central region.^2^ In contrast, less deprived municipalities were concentrated in the San Juan metropolitan area. This suggests that residents living in municipalities with lower SEPs may lack access to healthcare services and health-promoting resources due to economic, environmental, and physical barriers that impact health and increase the risk of mortality. However, these findings are conditional based on the assumptions made regarding area-based socioeconomic status. Better inference of neighborhood effects would require a more nuanced approach on how specific constructs of the neighborhood environment are measured (e.g., census tract vs. census block group) and how they influence health and the risk of mortality (16).

Recent efforts have been made to collect data on neighborhood-level attributes and link them to longitudinal population-based surveys [e.g., the Health and Retirement Study Contextual Data Resource (HRS-CDR)] (17). These linked data have allowed researchers to assess the influence of neighborhood characteristics on the health of older adults in the US. However, these data only include the contiguous US and exclude Alaska, Hawai'i, and the five permanently inhabited US territories, including Puerto Rico. Because of significantly differing historical and political contexts, and widely ranging structural factors between Puerto Rico and the US mainland, it is not appropriate to apply current knowledge on neighborhood health effects based on studies conducted in the contiguous US to Puerto Rico. In addition, despite Puerto Rico's status as an unincorporated US territory, its social and economic contexts are more like Latin American and Hispanic-Caribbean countries than the US, which may lead to substantially different risk factors for poor health and mortality.

In this study, we aim to highlight multilevel perspectives and analyses of social determinants of health among older adults residing in Puerto Rico. We address a gap in the literature by using longitudinal data from the Puerto Rican Elderly Health Conditions Project (PREHCO) linked with 2000 US Census data to (1) examine the types of neighborhood environments older Puerto Rican adults reside in and, (2) explore the association between neighborhood environment and all-cause mortality.

## 2. Background

It is widely recognized that physical and social environments influence health behaviors, health outcomes, and mortality in the US. Although the neighborhood environment affects the health of people of all ages, the effects of the neighborhood environment may be accentuated among older adults as they are more likely than younger adults to have spent decades in the same community, have decreased physical mobility and cognitive functioning, and rely more on community resources for social integration and support (18). The combination of these factors may result in an early onset of age-related diseases (19), reduced life expectancy (20), and an increased risk of all-cause mortality (21–23). Notably, a vast array of research has shown that individuals residing in neighborhoods with greater deprivation have poorer health behaviors (24), lack access to preventive health services (25), are exposed to chronic stress and pollutants (26), experience greater biological weathering (27), have worse health outcomes (28), and experience higher mortality rates (29). In many of these studies, neighborhood deprivation is based on socioeconomic contextual variables or indices related to income, education, employment, and housing, typically at the census tract level. Although these socioeconomic indicators have different meanings for older adults, it is noteworthy that the influence of socioeconomic deprivation persists in the oldest ages (30). Indeed, several studies suggest a cumulative effect of disadvantage across the lifespan that results in poor health and an increased risk of mortality (31). However, there is limited knowledge of how these multilevel processes influence population health and mortality in Puerto Rico due to the lack of data infrastructure to support these inquiries.

### 2.1. Neighborhood socioeconomic context

Various theoretical perspectives and conceptual frameworks have been put forth to explain why the neighborhood socioeconomic context (NSEC) plays a vital role in poor health outcomes and mortality risk. For instance, the ecological framework with a life course perspective would suggest that individuals living in disadvantaged NSECs are more likely to have a low socioeconomic position themselves due to constrained opportunity structures (22, 32, 33). Individuals who spend their early life in lower-income neighborhoods have less access to quality education than their peers residing in higher-income communities. This limits opportunities to obtain higher levels of education and marketable job skills and reduces lifetime earnings (34, 35). Thus, the importance of neighborhood context as a fundamental cause of mortality cannot be overlooked (36), particularly given the vast literature documenting how education shapes access to resources that promote better health and an individual's exposure to multiple health risks (37).

Another theoretical consideration is the systemic perspective, which infers that the NSEC affects the social, service, and physical environments of communities shared by residents. Namely, neighborhoods characterized by low socioeconomic levels are linked to underinvestment in health-promoting resources, such as lack of green and recreational spaces, adequate public transportation, affordable and high-quality grocery stores, and access to medical and social services (23). For example, individuals residing in high-poverty neighborhoods are less likely to have access to recreational opportunities to walk and exercise and are more likely to live in food swamps^3^ (38, 39). Not being able to engage in healthy behaviors due to these structural challenges can increase the likelihood of early disease onset, reduce active life expectancy (e.g., physical mobility), and increase the risk of mortality. Overall, the emphasis of the NSEC on health is important from a public health perspective since resource-poor environments can be potentially addressed through community-level interventions, including investments in public education, transportation, expansion of door-to-door services (e.g., Meals on Wheels), and affordable and quality housing to name a few.

Although research has overwhelmingly demonstrated that the NSEC is a crucial determinant of health, other neighborhood-related factors interplay with the NSEC, such as a neighborhood's age structure, racial composition, residential stability, and family structure that shape opportunities and health-enhancing resources made available for residents across communities. We provide a summary of how each of these neighborhood-level determinants potentially influences health outcomes and the risk of mortality.

### 2.2. Neighborhood age structure

The age structure of a neighborhood may be particularly important to older adults who age in place as it may influence the provision of health services and facilities (including Medicaid reimbursements), perceptions of neighborhood safety, and opportunities for social engagement (40). Previous research has shown that neighborhoods with a high concentration of older adults are associated with better health among older adults, including those who are socioeconomically disadvantaged (41, 42). Evidence suggests that the presence of older adults in the community facilitates social integration and cultivates social ties, mutual support, social cohesion, and perceived safety (43), which is independently associated with various population-level health outcomes, including mortality (44). Several pathways have been hypothesized on how aspects of the social environment may influence health and mortality, including the impact of health behaviors and physiology (e.g., allostatic load) (44, 45). Specifically, individuals with positive social ties are less likely to engage in smoking and drinking and are more likely to receive preventive health screenings (e.g., cancer screenings). In contrast, socially isolated individuals are more likely to have weakened immune function, cardiovascular disease, and cognitive impairment. Older adults with chronic health conditions, disabilities, who live alone, and have reduced social networks are at an increased risk of social isolation, which has been shown to negatively impact health and mortality.

With the population of Puerto Rico is rapidly aging—due to a combination of outmigration among younger cohorts of adults, declining fertility, and increased longevity—these demographic changes will challenge the ability of Puerto Rico and local communities to meet the growing demands of older adults, including care and quality of life, that may further strain the collective (and scarce) resources available (1, 3, 5, 46). Specifically, increases in poverty and declining economic conditions across the archipelago, changes in the family structure, and the limited availability (and proximity) of individuals and/or services to provide long-term care for older adults in Puerto Rico (due in part to out migration of family and professionals) may result in poor health and an increased risk of mortality. Older Puerto Ricans, cognizant of these social realities have expressed concerns with loss of family cohesion and intergenerational support due to their children's search of economic opportunities outside of Puerto Rico (47). This suggests that places in Puerto Rico that have a larger concentration of older adults, particularly in rural areas, may not have the resources necessary for older adults to successfully age in place.

### 2.3. Neighborhood racial composition

Neighborhood racial composition has been shown to be associated with poor health and an increased risk of mortality among older adults due in part to exposure of institutionalized and systemic anti-Black racism across the life course (48–50). A large body of research shows that Black (including African American and Afro-Latino) individuals in the US overwhelmingly reside in residentially segregated neighborhoods that are characterized by concentrated economic disadvantage, which is often associated with disinvestment of municipal resources (e.g., high-quality medical care), poorly maintained infrastructures (e.g., sidewalks and green spaces), and densely populated and subpar housing quality (51–53). These conditions stem from racial capitalism and environmental racism that intentionally create the underdevelopment of non-White spaces (54). The purposeful underdevelopment of these communities results in unequal exposure to contextual health-related risks that over time exact wear and tear on the body, which contributes to a process of “weathering,” leading to physiological dysregulation, the early onset of disease and disability, and ultimately mortality (55).

Although Puerto Rico appears to have a more flexible attitude toward race (i.e., the concept of “racial democracy”) than the US, there is ample evidence documenting that racial minorities, immigrants (e.g., Dominican immigrants), and phenotypically dark-skinned individuals in Puerto Rico are stigmatized, discriminated against, and experience more socioeconomic disadvantage than their more socially advantaged counterparts (14, 56–59). Notably, Black communities in Puerto Rico^4^ are largely located along the coastal regions of the Puerto Rican archipelago—a legacy of plantation slavery—and are regions that exhibit lower levels of education, lower median household income, lower median housing values, and higher rates of poverty and unemployment relative to predominantly White communities in Puerto Rico (60). Indeed, for Black Puerto Ricans, systemic and institutional racism across generations and across the lifespan have led to the inequitable access of social, educational, and material resources that have direct (e.g., access to health care) and indirect (e.g., stress and psychosocial resources) effects on health and mortality.

A community-based study of Puerto Rican adults aged 25–55 years in Guayama, Puerto Rico (a southeastern coastal town) found that respondents that are culturally defined as *negro* (Black) have higher systolic blood pressure (SBP) and diastolic blood pressure (DBP) than those who are classified as *blanco* (White) or *trigueño* (racially mixed)^5^ (61). Additionally, Black Puerto Ricans who occupy higher socioeconomic status (SES) positions exhibit higher SBP and DBP relative to their Black counterparts in low SES contexts (61). The authors posit that Black Puerto Ricans' chronic exposure to institutional and interpersonal discrimination may be linked to their adverse cardiovascular responses (i.e., high blood pressure). Thus, deeply embedded, and multiple dimensions of racism in Puerto Rico are associated with the pronounced residential segregation of Black Puerto Ricans that results in constrained access to resources and opportunities which affect health and mortality.

### 2.4. Neighborhood residential stability

Living in residentially stable neighborhoods is theorized to promote the health and wellbeing of its residents as it facilitates the development of interpersonal bonds and ties (i.e., social cohesion) that individuals can draw on in times of need (i.e., social support) and may encourage healthy behaviors, and extend longevity. However, a study by Ross et al. (62) found that residential stability was only associated with enhanced psychological wellbeing among residents in affluent neighborhoods. In contrast, residential stability did not benefit the mental health of residents in impoverished communities. Ross et al. posit that living in a poor, stable neighborhood does not confer mental health advantages since residents of these environments do not have the instrumental and material resources needed to mitigate the high levels of disorder in their communities. For example, the chronic stress associated with living long-term in a neighborhood where the streets are dirty, noisy, and dangerous repeatedly activates the stress response, which can contribute to blood pressure and brain changes associated with mental and physical health outcomes (63). Thus, the effects of residential stability need to be considered in the context of a neighborhood's economic resources available.

Data from the U.S. Census and Puerto Rican Community Surveys show that Puerto Rican have high residential stability (64); however, no study, to our knowledge, has examined whether neighborhood-level variation in residential stability is beneficial or detrimental to the health of older adults in Puerto Rico. The scant research that does exist on island-born Puerto Ricans residing in the mainland U.S. has shown that living in ethnically dense, low NSECs reported worse physical health than island-born Puerto Ricans living in other types of NSECs (65). Individuals residing in ethnic enclaves tend to share common sociocultural characteristics (e.g., language and cultural background) and have strong social ties with community members, which have been found to be beneficial for health and mortality. However, enclaves that are formed involuntarily due to housing discrimination may not offer opportunities necessary for economic development at the individual and community levels. Given the high rates of poverty across the archipelago, we can infer that residential stability may not confer health benefits for Puerto Ricans who are living in disadvantaged NSECs.

### 2.5. Neighborhood family structure

Research on the association between neighborhood family structure and mortality is scarce; however, neighborhood family structure is related to the formation of social ties, which has been shown to have a robust association with extended longevity (66). For example, residents in neighborhoods with high family dissolution (e.g., single-parent households) have lower participation rates in formal voluntary organizations and local affairs. These forms of participation provide opportunities for individuals to integrate within the larger community—additionally, neighborhoods with a high percentage of individuals living alone present opportunities for crime. Individuals who live alone are more likely to go outside alone, which increases the likelihood of a targeted crime (e.g., robbery). These incidents are more likely to instill perceptions of neighborhood disorder that may contribute to the dissolution of social ties and an increased risk of mortality.

Traditionally, Puerto Ricans are very family oriented, embody familism,^6^ and their families encompass extended and non-blood relatives (e.g., godparents and informally adopted children). The traditional structure of family dynamics in Puerto Rico has historically benefited older family members who often rely on family-based care. Recent research shows that intergenerational co-residence (e.g., children living with their older parents) is associated with increased functional and health support among older adults in Puerto Rico (67). However, the outmigration of younger Puerto Ricans to the US mainland, has led to a significant reduction in the number of family members available to provide care for older adult family members. Moreover, with increasing numbers of Puerto Ricans migrating in search of economic and educational opportunities, we can expect a higher risk of social isolation and lower social participation among older adults, which may be detrimental to mental and physical health (3). Thus, we can expect that communities in Puerto Rico with a high proportion of older adults that live alone and have a high proportion of single-parent households may be associated with worse health and an increased risk of mortality.

#### 2.5.1. The present study

There is compelling theoretical and empirical evidence illustrating how various dimensions of the neighborhood environment co-occur and/or interact to influence the risk of mortality. Given the limited knowledge on the types of residential environments that older Puerto Ricans reside in, it is important to characterize the places where they live based on the factors discussed above. Previous research has shown that using latent class (or profile) models offers an efficient and statistically robust means of summarizing many indicators that constitute neighborhood risks and resources that are not captured by continuous scales or indices (68, 69). We intend to employ this method to classify how various neighborhood characteristics cluster together to create distinct neighborhood typologies that capture risk for all-cause mortality.

## 3. Materials and methods

### 3.1. Data

#### 3.1.1. Individual-level data

This study used data from the Puerto Rico Elderly Health Conditions Project (PREHCO), a representative longitudinal cohort study of community-dwelling Puerto Ricans aged 60 and older residing on the archipelago's main island that began in May 2002, with follow-up interviews completed in 2006–2007 and 2021–2022 (the data and documentation are not yet publicly available) (70). Response rates for the first two waves of PREHCO are high (>90.0%). The 4,291 respondents included in the PREHCO baseline sample were derived from a multistage, stratified sample of older adults, including oversampling in regions heavily populated by Afro-descendant individuals (e.g., residents in Loíza) and individuals over 80 years of age. Face-to-face interviews were conducted with each respondent in Spanish or with a proxy if a respondent had cognitive limitations. Additional information on the study and its design is provided elsewhere (71–73).

PREHCO obtained mortality information on respondents using a combination of the National Death Index (NDI) mortality data and PREHCO-identified deaths using reports by family members or the Puerto Rican death registry. Respondents were matched to the National Death Index (NDI) from their first PREHCO interview in 2002–2003 to December 2020, using the available matching variables in the PREHCO study, including social security number (SSN), name (first, middle, father's last name and/or mother's last name), birth date (month and year), and sex (female or male). We would like to note that many Puerto Ricans use two surnames, which adds to the difficulty in NDI matching. Thus, the investigators examined different combinations of respondents' last names to increase the likelihood of a positive match for those with two last names. Additional deaths were identified through November 2021 using family reports or the Puerto Rican death registry. The data file comprising the currently restricted PREHCO mortality database contains the PREHCO respondent's case identification number, the mortality status of the respondent (presumed dead or alive), year of death, month of death, day of death (for some), and cause of death (for most respondents). Two thousand eight hundred and thirty-two all-cause presumed deaths were identified from the cohort of 4,291 PREHCO respondents.

#### 3.1.2. Neighborhood-level data

Data on baseline neighborhood characteristics were constructed from the 2000 Decennial US Census at the block-group level downloaded from Social Explorer and were linked with the PREHCO data (74). Census block groups typically include 600 to 3,000 people and is the smallest geographical unit for which the US Census Bureau publishes sample data. PREHCO respondents were linked to their affiliated census block group by linking their records in the public-use PREHCO to the restricted-use PREHCO geographic data file. These data were then merged with the 2000 US Census data using Federal Information Processing Standard (FIPS) codes to link the files. Out of the 2,477 unique census block group identifiers for Puerto Rico in 2000,^7^ we identified 233 unique census block groups in which PRECHO respondents resided at the time of the baseline interview, with 1–47 observations in each block group.

#### 3.1.3. Sample selection

The baseline PREHCO cohort sample consisted of 4,291 unique respondents aged 60 and older. Given the design of the present study, we focused on individuals who were able to complete the full interview at baseline (*n* = 3,713). Respondents that needed proxies to do the interview were not asked health-related questions relevant to the present study (*n* = 578). Furthermore, we excluded respondents (*n* = 24) in neighborhoods with <5 individuals in any given block group to minimize statistical bias (75). Lastly, we excluded ~6% of participants (*n* = 220) due to missingness on baseline covariates. The variables with the highest prevalence of missing values were body mass index (BMI; 5%) and receipt of government-related income and services (1%). The final analytical sample included 3,469 participants.

Participants excluded from the analytical sample were more likely to be older (76.8 vs. 70.3 years), less likely to be married or partnered (38.8 vs. 53.2%), reported lower levels of education (6.1 vs. 8.3 years), and were more likely to receive government-related income and services (see Supplementary Table 1). Additionally, excluded participants were less likely to be obese, current smokers, and physically active. Excluded participants were also more likely to report chronic conditions and disability. We caution readers that the health profiles of excluded participants may be underestimated since proxy interviews were not asked all of the health-related questions. Thus, our analytical sample of community-dwelling older Puerto Ricans is relatively healthier than the general population of older adults in Puerto Rico. A detailed scheme showing the exclusion criteria and the analytic sample inclusion is provided in Supplementary Figure 1.

Additionally, given that measures included in our analysis are time varying, we briefly describe changes in sample characteristics for Wave 2 of PREHCO. From our analytical sample of 3,469 participants identified in Wave 1, 941 respondents (27%) did not have information reported in Wave 2 relevant to the analysis, including 226 proxy interviews, 27 respondents that became institutionalized, 319 that were lost at follow-up, and 369 respondents that were reported dead. To keep all respondents in our analysis, we conducted multiple imputation using chained equations (MICE) for missing data at Wave 2 using the mi suite of commands in Stata (76). We used the distribution of the observed data from Waves 1 and 2 to estimate a set of plausible values for the missing data in Wave 2. We then used Bodner's approach of generating the number of imputed data sets equivalent to the total percent missing and Rubin's rule for combining across data sets (77–82).

### 3.2. Measures

#### 3.2.1. Mortality

The outcome of interest is all-cause mortality from May 2002 to November 2021. We calculated the time to censoring or death from the year of the interview to the year of death or censoring. For those who did not die in the interval, the censoring date was November 2021. We used years as the time metric.

#### 3.2.2. Individual-level characteristics

Three groups of individual-level variables were considered as potential confounders in examining the role of neighborhood context and all-cause mortality—demographic, socioeconomic, and health characteristics.

##### 3.2.2.1. Demographic variables

Age is measured in continuous years. We also included an age squared term, so we can model more accurately the effect of age rather than assuming the effect is linear for all ages. Sex was dichotomized as male or female. Marital status was dichotomized as married or partnered vs. never married, widowed, separated, or divorced. A dichotomous indicator for whether the respondent had moved from their main residence reported at baseline was also included.

##### 3.2.2.2. Socioeconomic variables

Educational attainment is measured as continuous years of education completed. Given that PREHCO has limited variables for assessing individual income (e.g., not having combined household annual income or poverty thresholds) and wealth (e.g., not having a standardized measure of all assets and debt), we used indirect measures of income, including whether a respondent reports having difficulty paying for their daily necessities (categorized as never, sometimes, and often), whether they receive income from social welfare or the department of the family^8^ (yes/no), whether they receive income from the nutritional assistance program (yes/no), and whether they have government-sponsored health insurance (excluding Medicare; yes/no). Given the strong association between individual-level socioeconomic position and mortality, it is crucial to adjust for individual socioeconomic measures to ensure the validity of neighborhood-level factors (83).

##### 3.2.2.3. Health characteristics

We included indicators related to the morbidity process such as health behaviors, health conditions, and disability (84). Health behaviors included obesity, current smoking status, and physical activity. Dichotomous indicators were used to classify respondents as obese (i.e., body mass index of ≥30 kg/m^2^), for whether the respondent reported being a current smoker at the time of the interview (yes/no), and whether they engaged in either moderate or vigorous physical activity at least three times per week (yes/no).

Health conditions included cardiometabolic diseases, other chronic conditions, and severe depression. Cardiometabolic diseases were a sum of whether the respondent self-reported heart problems (e.g., coronary heart disease, congestive heart failure, and heart attack), stroke, hypertension (including medication use) and diabetes (including medication use), ranging from 0 to 4. Other chronic conditions were a sum of self-reported cancer, lung disease, and arthritis, ranging from 0 to 3. We used the geriatric depression scale in its 15-item version (GSD-15) to classify respondents as having severe depression (85). Possible scores range from 0 (no depression) to 15 points (severe depression). Respondents were classified as having severe depression if they scored ≥10 points.

Disability was based on whether a respondent reported limitations in activities of daily living (ADLs) and instrumental activities of daily living (IADLs). ADLs are a continuous measure ranging from 0 to 6 and included difficulty with bathing, eating, dressing, walking across a room, getting in and out of bed, and using the toilet (86). IADLs are a continuous measure ranging from 0 to 7 and included difficulty with using the telephone, managing transportation, buying food or clothing, preparing meals, doing household tasks, taking medications, and managing finances.

#### 3.2.3. Neighborhood-level characteristics

We included variables at the block group level that are theoretically related to and have been identified in previous studies as being associated with all-cause mortality. Neighborhood characteristics included 19 indicators related to the neighborhood constructs of socioeconomic status, household composition, minority status, and housing and transportation. These indicators included the proportion of the population living in a rural area,^9^ Black residents, residents aged ≥65 years, older adults living alone, residents that lived in the same house past 5 years (residential stability), residents with <9 years of education, residents aged ≥16 years unemployed, residents aged ≥16 years employed in management, professional, and related occupations, households with income ≥$40,000, households with interest, dividend, or rental income, households with public assistance income, residents below 150% of the poverty threshold, single-parent households with children <18 years of age, renter-occupied housing units, residents living in crowded housing units, occupied housing units without complete plumbing, occupied housing units without a telephone, occupied housing units without a motor vehicle, and homes valued ≥150 k.

### 3.3. Statistical analysis

A latent profile analysis (LPA) was conducted using the gsem feature on Stata to characterize the types of neighborhood environments that older Puerto Ricans resided in at baseline (88). LPA is a semi-parametric finite mixture model that identifies homogenous subgroups based on common characteristics, creating mutually exclusive and exhaustive latent classes. LPA sorts data using posterior probabilities that calculate the probability of membership in each latent class given. Unlike other agglomerative approaches, such as cluster analysis and factor analysis, LPA is a non-parametric statistical technique that relaxes assumptions about normality and linearity in the variables used in the analyses, making LPA ideal for analyzing neighborhood-level characteristics since they do not have normal distributions. We selected the class solution that best represented the data using a combination of model fit statistics, the interpretability of the classes that emerged, and sample size per class once combined with the PREHCO data. When comparing class solutions based on model fit statistics, generally, lower values of the Akaike Information Criterion (AIC) and Bayesian Information Criterion (BIC) are preferred (89); and entropy with values approaching 1, indicating a clear delineation of classes, are preferred (90).

Next, we described the characteristics of the PREHCO analytic sample by each neighborhood cluster that emerged. Means and percentages were calculated using the xtsum and xttab features in Stata to account for the multilevel design and repeat observations.

Lastly, we estimated hazard ratios (HRs) and 95% confidence intervals (CIs) for all-cause mortality by applying a multilevel mixed-effects parametric survival model with a Weibull distribution and Berndt–Hall–Hall–Hausman (BHHH) optimization algorithm using the mestreg feature in Stata. We modeled our data with a three-level hierarchical structure: respondents (level 1) nested within each wave (level 2) and census block groups (level 3). Time-to-event was defined as the elapsed time, in years, from the baseline interview to the date of death or the end of the study follow-up, whichever came first. When we fitted a model, we included the neighborhood clusters and controlled for individual-level demographic variables: sex, age, age squared, and marital status (Model 1). Next, we proceeded to add individual-level socioeconomic indicators: education, income from social welfare, income from the nutritional assistance program, and government-sponsored health insurance (Model 2). Lastly, we added individual-level health characteristics: obesity, smoking, physical activity, cardiometabolic conditions, other chronic conditions, severe depression, and disability (i.e., ADLs and IADLs; Model 3).

All data wrangling, visualization, and analyses were conducted in Stata/MP version 17.0 (91). The data were weighted using PREHCO-provided sampling weights to ensure the representativeness of the PREHCO survey and to account for the sampling design to get reliable statistical estimates. The study protocol was deemed exempt by the Institutional Review Board at Syracuse University.

## 4. Results

### 4.1. Neighborhood clusters derived from the LPA

Latent profile models were fit based on 19 block-group level indicators using the 2,477 observations (i.e., unique block groups) available in the 2000 US Census for Puerto Rico, ranging from two to seven classes. Based on the model fit statistics, sample size, and accounting for interpretability, we chose the five-class model as having the best fit for further analysis (see Supplementary Tables 2–7). A summary of the five-class solution of neighborhood clusters is presented in Table 1.

**Table 1:** Summary of latent classes based on the year 2000 census block groups in Puerto Rico.

		**Urban low deprivation**	**Urban low-moderate deprivation**	**Rural moderate deprivation**	**Urban high-moderate deprivation**	**Urban high deprivation**
	**All block groups**					
Probability (class)		0.079	0.323	0.059	0.470	0.068
Probability of						
Rural	0.055	0.000	0.005	0.679	0.029	0.000
Black	0.082	0.040	0.083	0.040	0.086	0.129
Adults ≥65 years of age	0.124	0.172	0.135	0.102	0.118	0.081
Older adults living alone	0.328	0.323	0.302	0.299	0.338	0.417
Lived in same house past 5 years	0.727	0.666	0.714	0.770	0.746	0.688
<9 years of education	0.259	0.072	0.175	0.382	0.321	0.336
Unemployed	0.207	0.061	0.137	0.265	0.244	0.404
Employed in management and professional occupations	0.252	0.516	0.300	0.208	0.196	0.143
Households with ≥ $40,000 income	0.147	0.490	0.201	0.067	0.078	0.040
Households with interest, dividend, or rental income	0.048	0.188	0.053	0.024	0.029	0.020
Households with public assistance income	0.205	0.044	0.117	0.290	0.249	0.425
Population living below 150% of the poverty threshold	0.262	0.076	0.153	0.349	0.314	0.569
Single-parent households with children <18 years of age	0.190	0.117	0.168	0.131	0.189	0.436
Renter-occupied housing units	0.288	0.226	0.249	0.190	0.271	0.751
Living in crowded housing	0.194	0.095	0.156	0.259	0.217	0.269
Homes without complete plumbing	0.054	0.011	0.025	0.080	0.071	0.094
Homes without a telephone	0.241	0.047	0.137	0.346	0.305	0.422
Homes without a motor vehicle	0.302	0.158	0.215	0.294	0.339	0.635
Homes valued ≥ $150,000	0.124	0.556	0.108	0.081	0.072	0.099
Number of census block groups	2,477	236	783	143	1,149	166

Due to rounding, not all values add to 1.0.

We labeled the first cluster *Urban Low Deprivation* (Class 1), representing 7.9% of census block groups in Puerto Rico (*N* = 236). This cluster was characterized by block groups that were almost all urban, had the lowest proportion of Black individuals present, the highest proportion of older adults present, very favorable socioeconomic conditions, stable family structure, and favorable housing features relative to the other classes.

The second cluster was labeled *Urban Moderate-Low Deprivation* (Class 2) and represented 32.3% census block groups in Puerto Rico (*N* = 783). This cluster was characterized by block groups that were like the previous neighborhood cluster but notably had lower socioeconomic conditions, family structures that were somewhat less stable, and less favorable housing conditions compared to the first neighborhood cluster.

We labeled the third cluster *Rural Moderate Deprivation* (Class 3), representing 5.9% of census block groups in Puerto Rico (*N* = 143). This cluster was characterized by block groups that were predominantly rural, had a low proportion of Black individuals present, the lowest proportion of older adults living alone, unfavorable socioeconomic conditions, stable family structure, and unfavorable housing conditions relative to previous classes.

We labeled the fourth cluster *Urban Moderate-High Deprivation* (Class 4), representing 47.0% of census block groups in Puerto Rico (*N* = 1,149). This cluster was characterized by block groups that were predominantly urban, a higher proportion of older adults living alone, less favorable socioeconomic conditions, family structures that were less stable, and less favorable housing conditions relative to previous classes.

The final cluster represented 6.8% of census block groups in Puerto Rico (*N* = 166) and was labeled *Urban High Deprivation* (Class 5). This cluster was characterized by block groups that were urban, had the highest proportion of Black individuals present, the lowest proportion of older adults present yet the highest proportion of older adults living alone, very unfavorable socioeconomic conditions, unstable family structure, and unfavorable housing conditions relative to the other classes.

**Figure 1:**
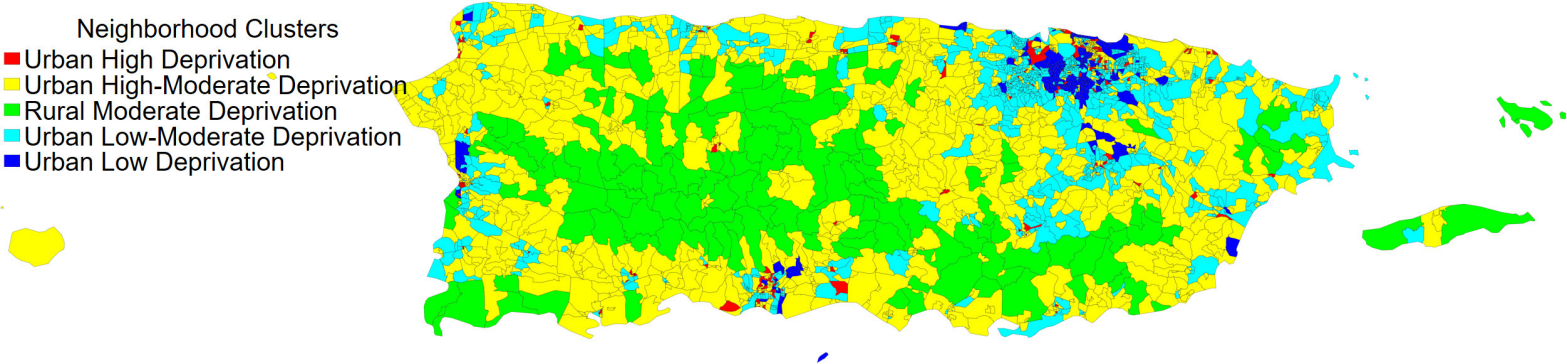
The distribution of neighborhood clusters by year 2000 census block groups in Puerto Rico. Data source: 2000 U.S. Decennial Census.

To better contextualize where these neighborhood clusters are geographically located in Puerto Rico, we provide a map of the neighborhood clusters identified in Puerto Rico by census block group (Figure 1). Neighborhoods that were classified as *Urban Low Deprivation* and *Urban High Deprivation* were mainly found in the municipalities of San Juan (the largest municipality), Ponce (the largest municipality outside the San Juan area), and Mayagüez (the largest municipality on the west side of the island). Neighborhoods characterized as *Urban Low-Moderate Deprivation* tended to be clustered outside larger municipalities (e.g., outside of San Juan). Neighborhoods characterized as *Urban High-Moderate Deprivation* and *Rural Moderate Deprivation* were distributed across the archipelago. Notably, neighborhoods in the *Rural Moderate Deprivation* cluster tended to be in the mountainous regions of the archipelago (i.e., the central part of Puerto Rico).

### 4.2. Characteristics of older Puerto Ricans by neighborhood cluster

The summary statistics of the PREHCO study sample by neighborhood cluster are presented in Table 2. We find that PREHCO respondents who resided in neighborhoods classified as *Urban Low Deprivation* (Class 1; *n* = 224), *Urban Low-Moderate Deprivation* (Class 2; *n* = 1,153), and *Rural Moderate Deprivation* (Class 3; *n* = 153) had a lower proportion of deaths over the study period relative to those residing in more disadvantaged neighborhood contexts. Older Puerto Ricans residing in the most advantaged neighborhood contexts included a higher proportion of female respondents, were older, less likely to move residences between waves, more educated, did not suffer from economic deprivation, and were less likely to report cardiometabolic conditions and disability. In contrast, respondents residing in the *Urban High Deprivation* (Class 5; *n* = 281) cluster had a higher proportion of individuals who died over the study period. The composition of this neighborhood cluster included a lower proportion of females, were younger, were the least likely to be married or partnered, more likely to move residences between waves, were less educated, suffered from economic deprivation, and were more likely to be classified with severe depression. Respondents in the *Rural Moderate Deprivation* (Class 3; *n* = 153) and *Urban High-Moderate Deprivation* (Class 4; *n* = 1,658) neighborhood clusters had similar demographic compositional profiles. However, respondents in the *Rural Moderate Deprivation* cluster had the lowest years of education attained relative to the other neighborhood clusters and had worse socioeconomic profiles relative to the *Urban High-Moderate Deprivation* cluster. Moreover, respondents in the *Rural Moderate Deprivation* cluster had relatively healthier behavioral profiles (e.g., more physically active, and lower proportion of obese individuals and current smokers) compared to the *Urban High-Moderate Deprivation* cluster.

**Table 2:** Characteristics of observations included in multilevel analysis by neighborhood cluster, PREHCO 2002–2007.

		**Neighborhood cluster of residence**				
		**Urban low deprivation**	**Urban low-moderate deprivation**	**Rural moderate deprivation**	**Urban high-moderate deprivation**	**Urban high deprivation**
	**Full sample**	**Class 1**	**Class 2**	**Class 3**	**Class 4**	**Class 5**
	% or mean ± SD					
**Individual-level demographic variables**						
Presumed dead	62.3	62.5	58.6	59.5	63.8	69.4
Female	59.7	70.5	61.9	51.0	57.1	61.9
Age (years)	73.4 ± 8.3	76.4 ± 9.1	73.1 ± 8.3	73.1 ± 8.3	73.1 ± 8.2	74.1 ± 8.3
Married or partnered	40.9	32.2	43.7	45.4	42.0	27.0
Moved from baseline residence	9.7	8.7	9.6	11.1	9.0	14.1
**Individual-level socioeconomic variables**						
Education (years)	8.1 ± 4.6	12.0 ± 3.8	9.4 ± 4.4	6.0 ± 3.9	7.0 ± 4.3	7.2 ± 4.4
Difficulty with daily needs being met						
Never	52.8	69.6	56.8	39.0	50.3	45.5
Sometimes	34.8	23.1	33.0	45.7	35.9	38.7
Often	12.4	7.3	10.2	15.3	13.8	15.9
Receives income from social welfare/department of the family	3.4	1.7	1.7	4.2	3.9	8.5
Receives income from the nutritional assistance program	29.0	10.6	19.6	48.4	33.8	43.6
Has government-sponsored health insurance	50.2	13.0	35.0	67.3	61.2	68.0
**Individual-level health variables**						
Obese (BMI ≥ 30 kg/m^2^)	27.4	27.9	29.5	21.3	26.5	26.5
Current smoker	6.9	4.5	5.0	7.2	8.5	6.9
Physically active	57.4	63.0	60.1	61.5	54.7	55.6
Cardiometabolic diseases (0–4)	1.1 ± 0.9	0.9 ± 0.8	1.1 ± 0.9	1.2 ± 1.0	1.1 ± 0.9	1.1 ± 0.9
Other chronic conditions (0–3)	0.4 ± 0.6	0.5 ± 0.6	0.4 ± 0.6	0.4 ± 0.6	0.4 ± 0.6	0.4 ± 0.6
Severe depression (GDS ≥ 10)	7.6	6.2	6.5	6.4	8.1	11.1
Activities of daily living (0–5)	0.3 ± 0.9	0.2 ± 0.8	0.3 ± 0.9	0.3 ± 0.8	0.4 ± 0.9	0.4 ± 1.0
Instrumental activities of daily living (0–5)	0.7 ± 1.3	0.6 ± 1.2	0.6 ± 1.2	0.7 ± 1.3	0.7 ± 1.3	0.8 ± 1.3
*N*	3,469	224	1,153	153	1,658	281

Weighted percentages and means; N's unweighted.

### 4.3. Association of neighborhood clusters with all-cause mortality

The results of the fitted multilevel survival models are summarized in Table 3. Hazard ratios (HR) are presented with 95% confidence intervals (CI). Hazard ratios >1 indicate that the mortality hazard is increasing, whereas hazard ratios <1 indicate that the mortality hazard is decreasing. The results of Model 1 (our base model) show that neighborhood clusters are associated with an increased hazard in all-cause mortality among older Puerto Ricans. Older adults that resided in the *Urban Low-Moderate Deprivation* [HR: 2.94; 95% CI (1.33, 6.49)], *Rural Moderate Deprivation* [HR: 2.60; 95% CI (1.10, 6.13)], *Urban High-Moderate Deprivation* [HR: 3.55; 95% CI (1.58, 7.94)], and *Urban High Deprivation* [HR: 5.59; 95% CI (2.24, 13.96)] clusters at baseline had higher mortality rates over the study period relative to the *Urban Low Deprivation* cluster. We also observed that female and married or partnered respondents had lower mortality rates over the study period, and that increasing age was associated with higher mortality rates, which is consistent with results from studies in high- and middle-income countries.

**Table 3:** All-cause mortality estimated from multilevel survival models of older Puerto Rican adults (*n* = 3,469).

**All-cause mortality**	**Model 1**			**Model 2**			**Model 3**		
	**HR**		**95% CI**	**HR**		**95% CI**	**HR**		**95% CI**
**Neighborhood-level variables**									
Neighborhood clusters (ref = urban low deprivation)									
Urban low-moderate deprivation	2.94	**	[1.33, 6.49]	2.30	*	[1.07, 4.97]	1.86		[0.92, 3.78]
Rural moderate deprivation	2.60	*	[1.10, 6.13]	1.83		[0.78, 4.30]	1.76		[0.80, 3.88]
Urban high-moderate deprivation	3.55	**	[1.58, 7.94]	2.80	*	[1.24, 6.33]	2.16	*	[1.02, 4.56]
Urban high deprivation	5.59	***	[2.24, 13.96]	4.74	**	[1.82, 12.30]	3.45	**	[1.39, 8.54]
**Individual-level demographic variables**									
Female (ref = male)	0.53	***	[0.42, 0.66]	0.53	***	[0.42, 0.67]	0.51	***	[0.39, 0.67]
Age	1.37	***	[1.24, 1.52]	1.47	***	[1.29, 1.66]	1.47	***	[1.29, 1.68]
Age squared	1.00	***	[1.00, 1.00]	1.00	***	[1.00, 1.00]	1.00	***	[1.00, 1.00]
Married or partnered	0.75	**	[0.63, 0.90]	0.78	**	[0.67, 0.92]	0.80	**	[0.69, 0.93]
Moved from baseline residence	1.36		[0.84, 2.21]	1.29		[0.80, 2.07]	1.20		[0.80, 1.78]
**Individual-level socioeconomic variables**									
Education (years)				0.97	*	[0.94, 0.99]	0.98		[0.96, 1.01]
Difficulty with daily needs being met (ref = often)									
Sometimes				0.90		[0.68, 1.18]	1.01		[0.78, 1.30]
Never				1.20		[0.85, 1.69]	1.31		[0.95, 1.80]
Receives income from social welfare/department of the family				0.98		[0.63, 1.50]	1.10		[0.72, 1.68]
Receives income from the nutritional assistance program				0.78	*	[0.63, 0.97]	0.88		[0.73, 1.06]
Has government-sponsored health insurance				1.36	**	[1.12, 1.65]	1.19		[0.99, 1.44]
**Individual-level health variables**									
Obese (BMI ≥ 30 kg/m^2^)							0.95		[0.80, 1.13]
Current smoker							1.53	***	[1.29, 1.81]
Physically active							0.72	**	[0.59, 0.89]
Cardiometabolic diseases (0–4)							1.19	***	[1.09, 1.29]
Other chronic conditions (0–3)							1.33	**	[1.09, 1.63]
Severe depression (GDS ≥ 10)							0.75		[0.53, 1.07]
Activities of daily living (0–5)							1.08		[0.96, 1.21]
Instrumental activities of daily living (0–5)							1.12	**	[1.03, 1.21]

* p < 0.05;

** p < 0.01;

*** p < 0.001.

HR, Hazard ratio; CI, Confidence interval.

Controlling for individual-level socioeconomic characteristics (Model 2) reduced the HR gradient of all the neighborhood clusters associated with all-cause mortality observed in Model 1. For example, adjusting for individual-level socioeconomic characteristics decreased the HR by ~32–37% for the *Urban Low-Moderate Deprivation, Urban High-Moderate Deprivation*, and *Urban High Deprivation* clusters but they remained significantly associated with all-cause mortality. Conversely, adjusting for individual-level socioeconomic characteristics reduced the *Rural Moderate Deprivation* cluster to non-significance [HR: 1.83; 95% CI (0.78, 4.30)]. Furthermore, our results indicate that higher levels of education and receiving nutritional assistance was associated with lower mortality over the study period, whereas reporting government-sponsored health insurance was associated with higher mortality over the study period.

Additionally controlling for individual-level health characteristics (Model 3) further reduced (changed) the HR for all the neighborhood clusters. The *Urban High-Moderate Deprivation* and *Urban High Deprivation* clusters exhibited an ~20–25% decrease (change) in the HR and were still significantly associated with all-cause mortality. For the *Urban Low-Moderate Deprivation* cluster, adjusting for individual-level health characteristics reduced the association to non-significance [HR: 1.86; 95% CI (0.92, 3.78)]. We also found that current smoker status, and reporting affirmative to individual items for cardiometabolic disease, other chronic conditions, and IADL limitations increased the hazard by 53, 19, 33, and 12%, respectively. In contrast, respondents that reported engaging in physical activity decreased the hazard by 28%.

**Figure 2:**
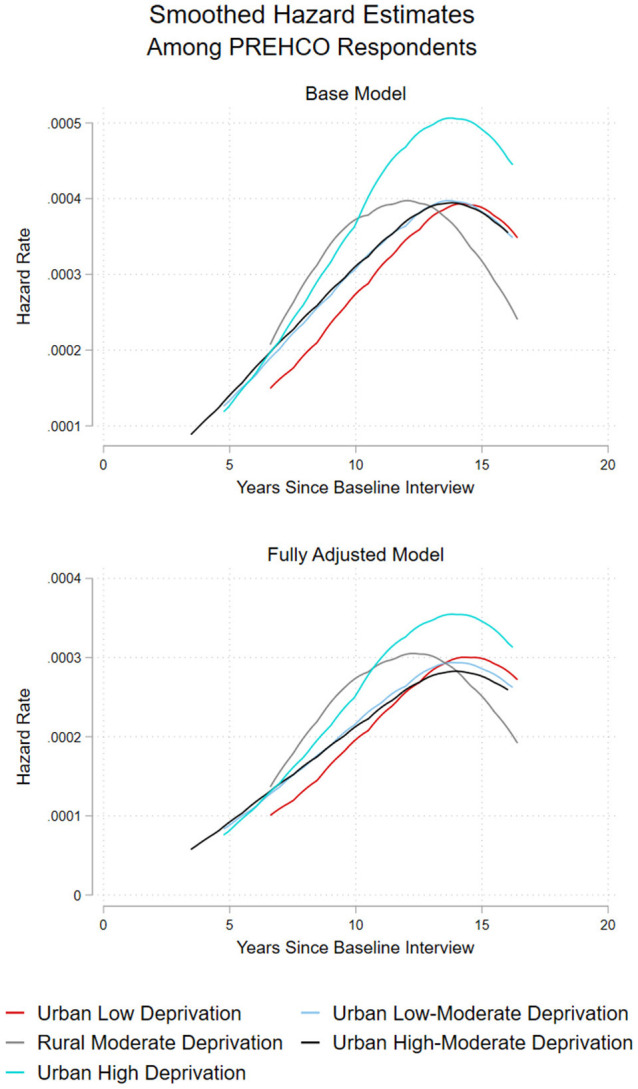
Smoothed hazard estimates of all-cause mortality by neighborhood cluster.

Post-estimation tests of coefficients from the final model indicated that older Puerto Ricans residing in the *Urban High Deprivation* cluster were at the highest risk of death over the study period compared to all the other neighborhood clusters in Puerto Rico. Smoothed hazard estimates of the risk of mortality by neighborhood cluster demonstrating this are shown in Figure 2.

## 5. Discussion

Using a population-based sample of community-residing individuals aged 60 and older in Puerto Rico, this study builds on prior literature documenting the effect of neighborhood environments on all-cause mortality among older adults. Using latent profile analysis to classify neighborhoods based on indicators related to the constructs of socioeconomic status, household composition, minority status, and housing and transportation resulted in five neighborhood clusters with varying patterns of social (dis)advantage: *Urban Low Deprivation, Urban Low-Moderate Deprivation, Rural Moderate Deprivation, Urban High-Moderate Deprivation*, and *Urban High Deprivation*. Our results show that older Puerto Ricans residing in neighborhoods classified as *Urban High Deprivation* and *Urban High-Moderate Deprivation* in Puerto Rico (over half of our analytical sample) exhibited an increased risk of mortality over the 19-year study period after adjustment for individual-level covariates. This suggests that a high concentration of unsupportive contexts for healthy aging increases the risk of premature death. This finding is consistent with other studies in the US and Latin America that have found exposure to disadvantaged neighborhood contexts to be a robust predictor of poor health outcomes and increased risk of mortality (27, 28, 92).

In contrast, residing in neighborhoods classified as *Rural Moderate Deprivation* and *Urban Low-Moderate Deprivation* was associated with all-cause mortality among older adults, however the association was attenuated once individual-level socioeconomic factors and health-related characteristics were accounted for, respectively. Previous research has shown that individuals residing in rural communities in the US tend to be less educated, have higher rates of poverty, and are less likely to have health insurance than those residing in urban communities (93). In the case of older Puerto Ricans that reside in *Rural Moderate Deprivation* contexts, our results indicate that the socioeconomic composition of individuals residing within these communities is a more important risk factor for all-cause mortality than the deprivation that exists at the community level. Furthermore, we can infer that older adults with socioeconomic or material advantages living in these communities can alleviate some of the adverse effects and exposures associated with these environments, which may be a family-level social selection mechanism that is unaccounted for in this study (94). It is possible that individuals with economic advantages residing in rural areas in Puerto Rico have been there for generations and chose to stay for reasons related to social, cultural, human, and financial capital (95). For older adults in *Urban Low-Moderate Deprivation* neighborhood contexts, we can infer that these individuals may self-select into neighborhoods with access to a wealth of social and material resources, such as having access to preventive health care services, and access to medical care that allows for the management of age-related diseases, which can increase longevity.

With the combination of rapid aging and compounding disasters in Puerto Rico, it is imperative to document and account for multilevel determinants of mortality for older adults across later stages of the life course. From a risk environment perspective, there is a need to delineate the environmental factors associated with the risk of mortality, such as the types of environments (e.g., physical, social, economic, and policy) and level of environmental influence (micro and macro), because understanding the places in which harm is produced and reduced offers a broader vision for intervention (96). For instance, a recent review found that the long-term impacts of air pollution, heavy metals, chemicals, ambient temperature, noise, radiation, and urban residential surroundings are associated with increased mortality (97). Since aging is an active response to “weathering,” we must consider how these environmental exposures are related to increases in inflammation, metabolic dysregulation, and genetic damage across the life span, increasing mortality risk. Specific to older adults, as their biological capacity declines with normal aging, the effects of deleterious environmental exposures may be exacerbated among individuals who enter the later stage of the life course with pre-existing health conditions and disabilities (98). Indeed, the biophysiological mechanisms underlying the neighborhood-mortality association are just beginning to be elucidated. Nonetheless, evidence does show that there are links between social factors, physiological dysregulation, and adult mortality (99). Future data collection efforts of older adults in Puerto Rico should include measures that represent multiple regulatory physiological systems (e.g., cardiovascular, metabolic, and immune) to comprehensively capture neighborhood influences on biology, and their contribution to health and mortality risks.

Considering Puerto Rico's socio-structural reality—including high levels of poverty, a deficient infrastructure, a fragile healthcare system, the dismantling of the public education system, and hazardous environmental exposures—a health disparities framework was established to reflect historical and sociocultural influences of the Puerto Rican population (100). We can draw on this framework to highlight how present disparities are rooted in historical, cultural, political, and economic factors that influence biology and behaviors and to illustrate the complex relationship between the neighborhood environment and mortality. For example, a recent study found that Puerto Rican adults residing in San Juan had multiple lifestyle risk factors and cardiometabolic conditions and recommended targeted efforts to improve the health care system and material resources among socially disadvantaged populations (101). While increasing material resources among older residents in the most disadvantaged neighborhood contexts may ease some of the challenges of aging in place, it does not get at the systemic causes of these challenges. For instance, the ports of Puerto Rico are controlled by mainland US agencies, leading to the high costs of (healthy) food on the archipelago (100). As a result, some older adults may forgo eating foods that may improve or better manage their health and decrease their mortality risk since they must make constrained choices on what to spend their limited incomes on. Thus, we recommend that policymakers, health care providers, and leaders across industries to (1) understand how individual health and mortality is embedded within larger social, cultural, structural, and historical contexts, and (2) make concerted efforts to reach out to residents living in disadvantaged community contexts to understand better what they need to successfully age in place in Puerto Rico. A study of residents in *La Perla* (an informal shantytown in San Juan with a high proportion of older adults) found that despite living in socially and economically disadvantaged residential environments, the residents reported high residential satisfaction because they built their neighborhood environment according to their community needs and have a network of support (102). This suggests that community engagement is essential to identify the health and social needs of Puerto Rican older adults and improve health in neighborhoods directly affected by inequities (103).

### 5.1. Limitations

Several limitations of this research should be acknowledged. First, we must recognize the physical resilience and robustness of Puerto Ricans who survived to older ages (i.e., aged ≥ 60 years) who were able to participate in the PREHCO study. Previous research has found that survival bias (or, selective survival) can attenuate associations between harmful exposures and age-related diseases, suggesting that the effects of harmful neighborhood environments may not be as pronounced among older adults and are likely underestimated (104).

Second, there are limitations associated with the operationalization of neighborhoods. We selected the smallest census unit for which we could obtain data—census block groups—to conceptualize neighborhoods in this study, an improvement from previous studies that have used census tracts as a neighborhood unit. However, recent research has emerged on the importance of activity spaces—defined as the places individuals encounter due to their day-to-day activities, which may not necessarily include their residential areas (105). Older adults may have activity spaces in more favorable or less advantageous environments relative to their residential settings that affect resources, exposures, benefits, and risks that have multifaceted effects on health and mortality. Future data collection efforts should consider capturing mobility and location information on older adults in Puerto Rico.

Third, using LPA to classify neighborhood clusters depends on the measures included to identify class types. Our findings may be biased by the exclusion of neighborhood characteristics important for distinguishing underlying neighborhood clusters, such as the built environment (e.g., availability of green spaces), availability of health care (e.g., number of physicians and number of facilities), neighborhood crime (e.g., violent offenses), and air pollution (e.g., PM_2.5_), which we lacked data on, to determine whether the identification of neighborhood clusters is improved. Nonetheless, we included multiple neighborhood variables across multiple neighborhood constructs that have been used in previous studies of all-cause mortality.

Fourth, as with any observational study, this study has unmeasured potential confounders that limit causal inference. For example, due to the limited measures related to income and wealth available in PREHCO, we could not examine if the influence of the neighborhood context differed by individual-level socioeconomic status (SES; e.g., low vs. moderate vs. high SES). Previous research has shown that death rates were higher among low SES individuals residing in high SES neighborhoods (92, 106). This suggests that there are potentially other subpopulations not captured in this study who are at higher risk for death.

Finally, we did not examine residential trajectories over time, which is especially relevant for Puerto Rico given the budget crisis, the great recession, the debt crisis, and Hurricanes Irma and María that may have resulted in increases in spatial inequality. PREHCO has publicly available data for two waves (2002–2003 and 2006–2007). The third wave of surviving respondents of PREHCO will be publicly available soon, and the fourth wave of data collection will begin later this year. These data will allow the creation of a longitudinal database to examine residential trajectories over time and their association with mortality.

Despite these limitations, our study makes several contributions on the role of neighborhoods on older adult mortality. First, we focus on older adults in Puerto Rico—a segment of the US and Latino population that is overlooked in US-based neighborhoods research and aging research more broadly. Second, we used latent profile analysis to summarize multiple indicators that constitute neighborhood risks and resources that are not captured by continuous scales or indices. Using this approach to identify neighborhood clusters associated with an increased risk of death in late life may help inform “upstream” points for structural interventions that can extend healthy years of life among older adults who have had adverse experiences throughout their life course. Third, we used longitudinal data to help establish causal inference. Using multilevel methods and longitudinal data, we assessed the temporal relationship of the association between the neighborhood context at baseline and 19-year all-cause mortality, controlling for possible confounders, allowing for more robust causal inferences. This investigation serves as a foundation to highlight a multilevel perspective of social determinants of health in Puerto Rico. Collectively, we must reframe the narrative on the aging process in Puerto Rico to understand the interplay that historical, environmental, social, behavioral, and biological factors have on health and mortality in this rapidly aging population. Through these efforts, we can identify opportunities to assess and improve the health and wellbeing of older Puerto Rican adults.

## Data availability statement

Publicly available datasets were analyzed in this study. This data can be found at: https://doi.org/10.3886/ICPSR34596.v1 for PREHCO and https://www.socialexplorer.com/ for the 2000 US Decennial Census.

## Ethics statement

The original two waves of the PREHCO study complied with all the IRB requirements at the University of Wisconsin-Madison and the University of Puerto Rico. The use of the NDI mortality data with the PREHCO study complied with IRB requirements at the University of Alabama-Birmingham. The use of the geographic data with the PREHCO study complied with IRB requirements at Syracuse University.

## Author contributions

CG conceptualized and designed the study, organized and conducted the statistical analysis, interpreted the results and findings, prepared all data visualization, and wrote the manuscript. MG assisted with the interpretation and validation of results and revised the manuscript critically for important intellectual content. MM created the mortality data for PREHCO for use in this study and revised the manuscript. MC assisted with the conceptualization of the study, revised the manuscript, and acquired financial support for PREHCO. All authors approve the submitted version of this manuscript and agree to be accountable for the content of the work.

## Footnotes

^1^Healthy aging here is defined as good health, high mental and physical functioning, and active involvement in life.

^2^The central region of Puerto Rico has many mountainous places and a higher proportion of individuals living in rural areas.

^3^Food swamps describe the combination of food deserts and a high-density of stores and restaurants that offer high-calorie fast food and junk food.

^4^Predominantly Black communities in Puerto Rico are located in the municipalities of Loíza, Yabucoa, and Patillas to name a few.

^5^Trigueño in English literally means “wheat colored” and is used to (1) describe a light-skinned person with a slightly tanned complexion; or (2) describe someone with European, African, and Native American heritage. Being classified as trigueño may be socially advantageous since it does not carry the stigma of blackness.

^6^Familism emphasizes obligation and duties of families to one another.

^7^Every census block group has a unique 12-digit FIPS code.

^8^The Puerto Rico department of the family specializes in individual and family social services.

^9^The US Census Bureau classifies urban/rural areas based on population thresholds, density, and land-use characteristics. Typically, rurality is designated as all population, housing, and territory not included within an urbanized area or urban cluster (≥2,500 people) (87).
