# Supplementary material for: The neighborhood context and all-cause mortality among older adults in Puerto Rico
Source: Front Public Health. 2023 Mar 9;11:995529. doi: 10.3389/fpubh.2023.995529 (PMC10034172; doi:10.3389/fpubh.2023.995529)

Supplementary Tables

Supplementary Table 1. Baseline Characteristics of the PREHCO Cohort by Sample Status, PREHCO 2002-2003.

	Excluded from Analytical Sample	Analytical Sample	
	n = 822	n = 3,469	
	% or Mean (±SD)		χ^2 or F test
**Individual-Level Demographic Variables**			
Female	56.2	55.5	n/s
Age	76.8 ± 10.1	70.3 ± 7.5	<.001
Married or Partnered	38.8	53.2	<.001
**Individual-Level Socioeconomic Variables**			
Education (years)	6.1 ± 4.7	8.3 ± 4.5	<.001
Daily Needs Being Met			
Never	47.5	52.7	n/s
Sometimes	35.4	35.2	n/s
Often	17.1	12.1	n/s
Receives Income from Social Welfare/Department of the Family	6.5	2.7	<.01
Receives Income from the Nutritional Assistance Program	32.4	21.4	<.001
Has Government-Sponsored Health Insurance	56.8	47.5	<.001
**Individual-Level Health Variables**			
Obese (BMI ≥30 kg/m^2)	19.4	28.0	<.001
Current Smoker	7.2	7.7	<.05
Physically Active	15.3	44.3	<.001
Cardiometabolic Diseases (0-4)	1.3 ± 1.1	1.1 ± 1.0	<.001
Other Chronic Conditions (0-3)	0.7 ± 0.7	0.6 ± 0.7	<.05
Severe Depression (GDS ≥10)	10.7	6.9	n/s
Activities of Daily Living (0-5)	1.0 ± 1.7	0.2 ± 0.7	<.001
Instrumental Activities of Daily Living (0-5)	1.5 ± 1.9	0.5 ± 1.2	<.001
**Neighborhood-Level Variables**			
Neighborhood Cluster of Residence			
Urban Low Deprivation	8.6	7.5	n/s
Urban Low-Moderate Deprivation	28.6	33.8	n/s
Rural Moderate Deprivation	5.3	5.2	n/s
Urban High-Moderate Deprivation	49.8	46.9	n/s
Urban High Deprivation	7.7	6.7	n/s

Note: Proxy interviews were not asked health-related questions.

Supplementary Table 2. Model Fit Indices for Latent Profile Analysis (LPA) Models for Year 2000 Census Block Groups in Puerto Rico.

Classes	Free Parameters	Log-Likelihood	AIC	BIC	Entropy
# Of Classes					
1	38	34230.26	-68384.52	-68164.23	1.000
2	58	39578.25	-79040.51	-78704.27	0.924
3	78	42541.44	-84926.88	-84474.69	0.890
4	98	44941.44	-89686.87	-89118.74	0.892
**5**	**118**	**46550.62**	**-92865.23**	**-92181.15**	**0.891**
6	138	47593.35	-94910.71	-94110.68	0.862
7	158	48309.58	-96303.16	-95387.19	0.838
Sample		2,477 Census Block Groups			

Note: AIC = Akaike Information Criterion

BIC = Bayesian Information Criterion

Indices for optimal model bolded

Supplementary Table 3. Summary of Latent Classes Based on 2000 Puerto Rico Census Block Groups.

	All Block Groups	Class 1	Class 2
Probability (class)		0.362	0.638
Probability of			
% Rural	0.055	0.007	0.083
% Black	0.082	0.072	0.088
% Adults ≥65 Years of Age	0.124	0.145	0.112
% Older Adults Living Alone	0.328	0.306	0.341
% Lived in Same House Past 5 Years	0.727	0.700	0.742
% <9 Years of Education	0.259	0.144	0.324
% Unemployed	0.207	0.117	0.258
% Employed in Management and Professional Occupations	0.252	0.356	0.193
% Households with ≥ $40,000 Income	0.147	0.271	0.077
% Households with Interest, Dividend, or Rental Income	0.048	0.084	0.028
% Households with Public Assistance Income	0.205	0.096	0.266
% Population Living Below 150% of the Poverty Threshold	0.262	0.130	0.338
% Single-Parent Households with Children <18 years of age	0.190	0.154	0.210
% Renter-Occupied Housing Units	0.288	0.245	0.313
% Living in Crowded Housing	0.194	0.138	0.226
% Homes without Complete Plumbing	0.054	0.020	0.073
% Homes without a Telephone	0.241	0.111	0.314
% Homes without a Motor Vehicle	0.302	0.198	0.361
% Homes Valued ≥ $150,000	0.124	0.208	0.076
Number of Census Block Groups	2,477	875	1,602
Number of PREHCO Respondents	4,291	1,486	2,805

Note: Due to rounding, not all values add to 1.0.

Supplementary Table 4. Summary of Latent Classes Based on 2000 Puerto Rico Census Block Groups.

	All Block Groups	Class 1	Class 2	Class 3
Probability (class)		0.509	0.085	0.405
Probability of				
% Rural	0.055	0.096	0.001	0.016
% Black	0.082	0.088	0.042	0.082
% Adults ≥65 Years of Age	0.124	0.111	0.171	0.132
% Older Adults Living Alone	0.328	0.346	0.321	0.308
% Lived in Same House Past 5 Years	0.727	0.742	0.663	0.720
% <9 Years of Education	0.259	0.340	0.073	0.195
% Unemployed	0.207	0.277	0.064	0.149
% Employed in Management and Professional Occupations	0.252	0.182	0.512	0.286
% Households with ≥ $40,000 Income	0.147	0.067	0.478	0.180
% Households with Interest, Dividend, or Rental Income	0.048	0.025	0.182	0.049
% Households with Public Assistance Income	0.205	0.287	0.046	0.134
% Population Living Below 150% of the Poverty Threshold	0.262	0.365	0.080	0.173
% Single-Parent Households with Children <18 years of age	0.190	0.219	0.121	0.168
% Renter-Occupied Housing Units	0.288	0.328	0.233	0.250
% Living in Crowded Housing	0.194	0.234	0.096	0.164
% Homes without Complete Plumbing	0.054	0.079	0.011	0.030
% Homes without a Telephone	0.241	0.337	0.050	0.160
% Homes without a Motor Vehicle	0.302	0.383	0.163	0.229
% Homes Valued ≥ $150,000	0.124	0.071	0.541	0.103
Number of Census Block Groups	2,477	1,247	206	1,024
Number of PREHCO Respondents	4,291	2,116	295	1,880

Note: Due to rounding, not all values add to 1.0.

Supplementary Table 5. Summary of Latent Classes Based on 2000 Puerto Rico Census Block Groups.

	All Block Groups	Class 1	Class 2	Class 3	Class 4
Probability (class)		0.508	0.346	0.081	0.066
Probability of					
% Rural	0.055	0.101	0.011	0.001	0.001
% Black	0.082	0.081	0.084	0.040	0.130
% Adults ≥65 Years of Age	0.124	0.116	0.134	0.172	0.079
% Older Adults Living Alone	0.328	0.333	0.306	0.323	0.419
% Lived in Same House Past 5 Years	0.727	0.750	0.715	0.666	0.685
% <9 Years of Education	0.259	0.332	0.180	0.072	0.333
% Unemployed	0.207	0.250	0.140	0.062	0.405
% Employed in Management and Professional Occupations	0.252	0.194	0.297	0.515	0.144
% Households with ≥ $40,000 Income	0.147	0.075	0.195	0.487	0.040
% Households with Interest, Dividend, or Rental Income	0.048	0.027	0.052	0.187	0.019
% Households with Public Assistance Income	0.205	0.258	0.121	0.044	0.424
% Population Living Below 150% of the Poverty Threshold	0.262	0.323	0.158	0.076	0.571
% Single-Parent Households with Children <18 years of age	0.190	0.184	0.168	0.118	0.441
% Renter-Occupied Housing Units	0.288	0.261	0.251	0.227	0.766
% Living in Crowded Housing	0.194	0.224	0.158	0.095	0.269
% Homes without Complete Plumbing	0.054	0.074	0.026	0.011	0.093
% Homes without a Telephone	0.241	0.314	0.143	0.048	0.421
% Homes without a Motor Vehicle	0.302	0.337	0.220	0.159	0.639
% Homes Valued ≥ $150,000	0.124	0.071	0.107	0.552	0.102
Number of Census Block Groups	2,477	1,240	839	196	202
Number of PREHCO Respondents	4,291	2,190	1,476	295	330

Note: Due to rounding, not all values add to 1.0.

Supplementary Table 6. Summary of Latent Classes Based on 2000 Puerto Rico Census Block Groups.

	All Block Groups	Class 1	Class 2	Class 3	Class 4	Class 5	Class 6
Probability (class)		0.380	0.077	0.147	0.037	0.301	0.058
Probability of							
% Rural	0.055	0.036	0.000	0.004	0.000	0.004	0.685
% Black	0.082	0.074	0.039	0.126	0.131	0.084	0.040
% Adults ≥65 Years of Age	0.124	0.108	0.173	0.140	0.066	0.136	0.102
% Older Adults Living Alone	0.328	0.308	0.321	0.429	0.388	0.301	0.299
% Lived in Same House Past 5 Years	0.727	0.763	0.668	0.677	0.721	0.712	0.770
% <9 Years of Education	0.259	0.315	0.071	0.325	0.344	0.169	0.384
% Unemployed	0.207	0.231	0.061	0.282	0.460	0.135	0.267
% Employed in Management and Professional Occupations	0.252	0.198	0.517	0.196	0.119	0.305	0.207
% Households with ≥ $40,000 Income	0.147	0.085	0.494	0.066	0.026	0.207	0.066
% Households with Interest, Dividend, or Rental Income	0.048	0.028	0.190	0.034	0.012	0.054	0.024
% Households with Public Assistance Income	0.205	0.241	0.044	0.276	0.489	0.113	0.291
% Population Living Below 150% of the Poverty Threshold	0.262	0.291	0.074	0.392	0.650	0.146	0.351
% Single-Parent Households with Children <18 years of age	0.190	0.167	0.115	0.279	0.505	0.168	0.131
% Renter-Occupied Housing Units	0.288	0.214	0.221	0.482	0.904	0.248	0.191
% Living in Crowded Housing	0.194	0.222	0.095	0.207	0.286	0.153	0.259
% Homes without Complete Plumbing	0.054	0.071	0.011	0.076	0.076	0.023	0.081
% Homes without a Telephone	0.241	0.295	0.046	0.332	0.447	0.131	0.347
% Homes without a Motor Vehicle	0.302	0.289	0.155	0.504	0.706	0.209	0.295
% Homes Valued ≥ $150,000	0.124	0.071	0.561	0.093	0.074	0.108	0.082
Number of Census Block Groups	2,477	930	186	357	91	728	185
Number of PREHCO Respondents	4,291	1,610	270	836	112	1,272	191

Note: Due to rounding, not all values add to 1.0.

Supplementary Table 7. Summary of Latent Classes Based on 2000 Puerto Rico Census Block Groups.

	All Block Groups	Class 1	Class 2	Class 3	Class 4	Class 5	Class 6	Class 7
Probability (class)		0.037	0.348	0.058	0.039	0.293	0.082	0.143
Probability of								
% Rural	0.055	0.000	0.039	0.688	0.000	0.005	0.003	0.003
% Black	0.082	0.129	0.073	0.040	0.031	0.086	0.059	0.126
% Adults ≥65 Years of Age	0.124	0.065	0.107	0.102	0.186	0.134	0.146	0.142
% Older Adults Living Alone	0.328	0.387	0.308	0.298	0.322	0.306	0.296	0.440
% Lived in Same House Past 5 Years	0.727	0.709	0.764	0.770	0.675	0.725	0.659	0.679
% <9 Years of Education	0.259	0.348	0.323	0.383	0.062	0.188	0.093	0.322
% Unemployed	0.207	0.455	0.238	0.267	0.054	0.145	0.085	0.282
% Employed in Management and Professional Occupations	0.252	0.117	0.193	0.207	0.567	0.280	0.442	0.198
% Households with ≥ $40,000 Income	0.147	0.026	0.080	0.066	0.552	0.179	0.381	0.066
% Households with Interest, Dividend, or Rental Income	0.048	0.011	0.026	0.024	0.248	0.048	0.109	0.034
% Households with Public Assistance Income	0.205	0.484	0.249	0.291	0.041	0.127	0.060	0.276
% Population Living Below 150% of the Poverty Threshold	0.262	0.643	0.301	0.351	0.064	0.160	0.099	0.392
% Single-Parent Households with Children <18 years of age	0.190	0.504	0.167	0.131	0.100	0.172	0.139	0.279
% Renter-Occupied Housing Units	0.288	0.905	0.212	0.191	0.205	0.245	0.249	0.488
% Living in Crowded Housing	0.194	0.294	0.226	0.259	0.074	0.162	0.122	0.203
% Homes without Complete Plumbing	0.054	0.087	0.074	0.081	0.008	0.027	0.013	0.073
% Homes without a Telephone	0.241	0.454	0.305	0.347	0.039	0.149	0.071	0.327
% Homes without a Motor Vehicle	0.302	0.710	0.293	0.295	0.152	0.222	0.165	0.504
% Homes Valued ≥ $150,000	0.124	0.073	0.069	0.082	0.716	0.090	0.307	0.093
Number of Census Block Groups	2,477	92	847	140	94	716	198	390
Number of PREHCO Respondents	4,291	112	1,515	175	185	1,288	214	802

Note: Due to rounding, not all values add to 1.0.

Supplementary Figures

Supplemental Figure 1. Selection of the PREHCO Analytic Sample.

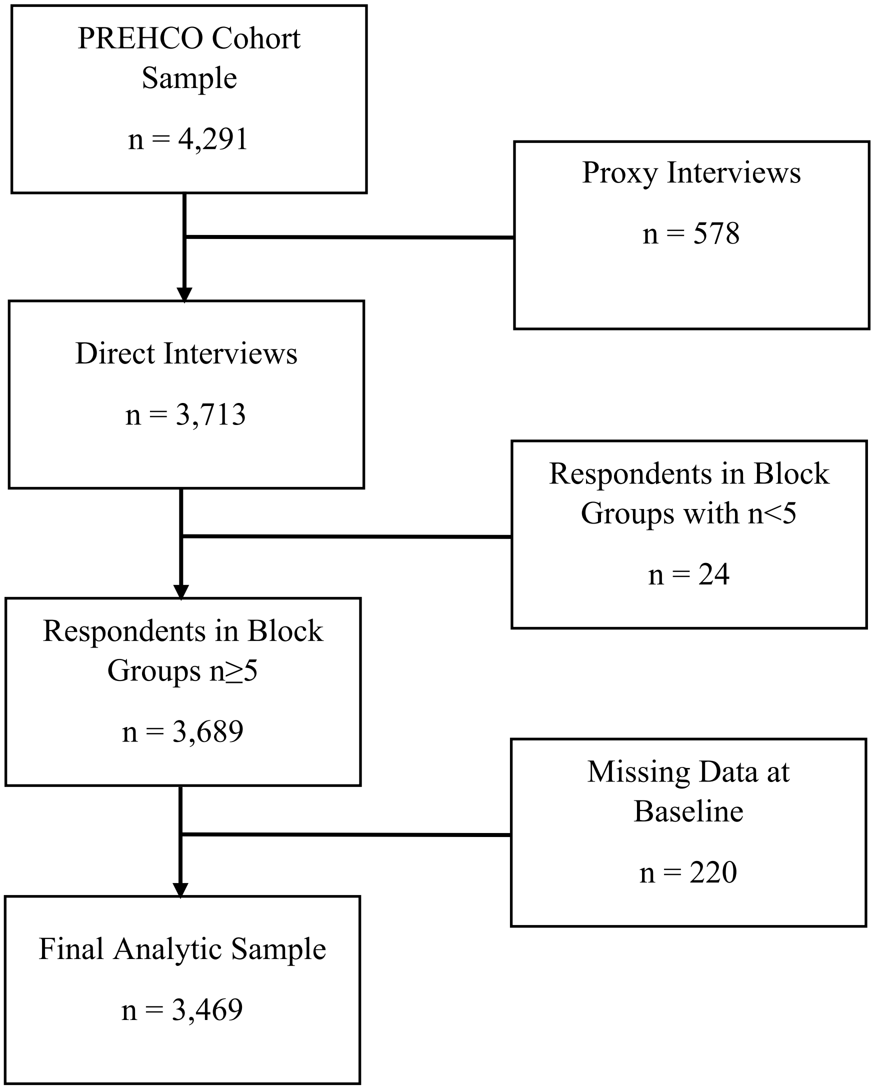

Supplement: Supplementary file 1 [file Data_Sheet_1.PDF]
